# Healthcare Providers’ Perceptions of Vulnerability to Domestic Sex Trafficking in Ontario: A Qualitative Study

**DOI:** 10.1177/11786329251348295

**Published:** 2025-06-24

**Authors:** Corinne Rogers, Soumyaa Veerakumar Subramanium, Rhonelle Bruder, Robin Mason, Janice Du Mont

**Affiliations:** 1 Women’s College Research Institute, Women’s College Hospital, Toronto, ON, Canada; 2 Dalla Lana School of Public Health, University of Toronto, Toronto, ON, Canada

**Keywords:** domestic sex trafficking, critical social theory, intersectionality, healthcare providers, vulnerability

## Abstract

**Background:**

Domestic sex trafficking is a prevalent health and human rights issue in Ontario, Canada. Although providers working in healthcare settings are uniquely positioned to identify and care for individuals who are sex trafficked, they may be hampered by a limited understanding of who is vulnerable to being sex trafficked and, thereby, fail to recognize those in need of support.

**Objectives:**

This qualitative study, part of a larger program of research, sought to apply critical social theory, and intersectionality to explore providers’ perceptions of who is vulnerable to domestic sex trafficking.

**Methods:**

Thirty-one healthcare providers of diverse identities and professional backgrounds were interviewed, using open-ended semi-structured questions, between November 2022 and February 2023. The interviews were analyzed using Braun and Clarke’s reflexive thematic analysis framework and organized by a modified Taxonomy of Vulnerability.

**Results:**

Three themes were generated: Traumatic history, social identities and relationships, and structural determinants. Providers consistently identified being female as a vulnerability to domestic sex trafficking. Few providers referenced the intersections of being female with other sociodemographic characteristics or acknowledged the complex ways in which larger systems have perpetuated the marginalization and inequitable status of some persons.

**Conclusion:**

The findings emphasize the urgent need to understand vulnerability as more than just an individual condition. Further, provider training must cultivate critical consciousness to recognize the contextual roots of vulnerability and how the role and socialization processes of larger systems in perpetuating vulnerabilities differently across individuals’ lives.

## Introduction

Domestic trafficking in Canada frequently involves sex trafficking^1^ Domestic sex trafficking refers to the recruitment and sexual exploitation of individuals with varying legal status entirely within a country’s national borders.^2^ Studies have shown that while sex-trafficked, up to 63% of individuals seek care through hospital emergency departments, while nearly 88% of individuals seek care through alternative health settings^3^ that include: community clinics, mental health clinics, reproductive clinics, pharmacies, shelters, and substance use centers.^4-12^ Yet, providers have reported finding it difficult to identify individuals who are sex trafficked for a variety of reasons including their perceptions being informed by biased media depictions, trafficking myths, and variable conceptualizations of vulnerabilities.^13-16^ Thus, despite seeking care, this does not guarantee that persons who are sex trafficked will receive appropriate support as healthcare providers find it difficult to identify them.^13^ To challenge misconceptions about who is vulnerable to domestic sex trafficked, it is important we explore providers’ current perceptions of who is vulnerable.

## Vulnerability

Meanings of vulnerability shift with changing socio-economic and political conditions. Traditionally, specific individuals, groups, or populations are labeled vulnerable when perceived and/or positioned outside of societal norms of being human.^17^ These societal norms are driven by “social motivations, necessitate conditional preferences for compliance, and reflect social pressure acknowledged through social feedback in specific situations,” (p. 2)^18,19^ such as social identities and status. Therefore, an individual is vulnerable when they are marginalized because of their social identity. Generally, vulnerability is linked to concepts “like harm, need dependency, care, and exploitation” (p. 2)^17^ and is often associated with “diminished agency or ability” (p. 1372).^20^ In contrast, vulnerability, framed through Vulnerability Theory, is understood to be a universal human condition that “put[s] all members of society on an equal grounding” (p. 48)^21^ and requires one’s “inescapable, life-long reliance on social institutions and relationships” (p. 1372).^20^ Recently, work on vulnerability theory also considers the role of social institutions within social relationships and responsibilities in understanding the concept.^20^ However, vulnerability theory is problematic when situated in the inherent universal biological conditions of being human, without directly addressing historical and systemic sources of oppression to question privilege, asymmetries of power, and responsibility.^22^

When we conceptualize vulnerability as a universal experience of the human condition, it necessitates an acknowledgment of the ways that asymmetries of power and systems of oppression manifest and render some groups vulnerable to exploitation.^17^ The Taxonomy of Vulnerability also conceptualizes the concept of vulnerability with its universality.^17^ The Taxonomy integrates the ontological and context-specific understandings of the concept, to categorize and distinguish different “sources and states of vulnerability” (p. 7).^17^ Further, the Taxonomy identifies responsibilities owed to the perpetuation of vulnerabilities.^17^ The taxonomy consists of three sources of vulnerability: “inherent, situational, and pathogenic” (p. 1).^17^ Inherent sources refer to sources of vulnerability universal to all humans as part of the human condition.^17^ These vulnerabilities are “independent of time, space, and conditions,” (p. 759)^23^ as they arise from our intrinsic needs, our “dependence on others and our affective and social natures” (p. 7).^17^ These vulnerabilities include love, safety, and hunger.^17^ Situational vulnerabilities are context specific and arise from “personal, social, political, economic or environmental conditions of individuals or social groups,” (p. 7)^17^ such as a public health crisis.^17^ These vulnerabilities have “a relation with time, space, and conditions” (p. 759).^23^ Finally, pathogenic vulnerabilities arise from a variety of ethically challenging situations, particularly “morally dysfunctional or abusive interpersonal and social relationships, sociopolitical oppression or injustice” (p. 9).^17^ Pathogenic vulnerabilities include those faced by an individual with varying cognitive abilities, dependent on others for their care and are abused by their caregiver.^17^

Most vulnerability studies focus on theoretical debates regarding citizenship, governance, resilience, and policy critiques, with few focusing on key informants’ viewpoints, such as service providers and clients.^24^ Although Schwarz et al used grounded theory to identify risk factors for labor or sex trafficking from service providers’ perceptions,^25^ and Hu used a feminist post-colonial framework to explore websites of organizations to identify risk factors that could make someone vulnerable to labor or sexual exploitation,^26^ neither explored providers’ perceptions of vulnerability specifically in the context of domestic sex trafficking, a critical gap.

## Providers’ Perceptions of Vulnerability

In exploring providers’ perceptions of the vulnerabilities of individuals through critical social theory and intersectionality, we can identify the systems responsible for these vulnerabilities. For example, in one study, providers from diverse sectors perceived “that most trafficked persons go in and out of services throughout their exploitation, including social services, housing assistance, educational systems, and medical assistance,” (p. 128),^25^ raising the question, “What do these various providers perceive as comprising vulnerability to domestic sex trafficking?” Knowing the answer to this question would advance the conceptualization of the concept of vulnerability. Consequently, this knowledge could further inform how we understand vulnerabilities to domestic sex trafficking perpetuated by systems and structures of power. Which, in turn, could lay the foundation for developing appropriate education and training for providers that support them to provide appropriate services and supports and challenge the systems and structures of power that perpetuate vulnerabilities to domestic sex trafficking.

## Theoretical Framework

Critical social theory, an “evaluative approach” (p. 7)^27^ of social justice theory, frames this study, as it encourages the examination of variation in individual lives, social practices, institutional structures, and cultural beliefs create vulnerabilities that can intersect to heighten the risk of domestic sex trafficking. These intersections are “greater than the sum of intersecting axes” (p. 140).^28^ If we understand these intersections as formed by vulnerabilities that are simply additive to each other rather than co-occurring, misapprehensions about these vulnerabilities can result. However, when we examine intersecting vulnerabilities of domestic sex trafficking critically—such as socioeconomic status, race, and gender as co-occurring rather than in addition to each other—we recognize the need to consider the complex and the nuanced and multifaceted ways in which these realities shape individual’s lives. Further, as a “political activity,” (p. 7)^27^ critical social theory considers the differing impacts of larger systems on individuals’ and groups’ lives by revealing and challenging power asymmetries. Thus, adopting an intersectional analytical approach in this study can “capture differences in individual lives, social practices, institutional arrangements, and cultural ideologies” (p. 2)^29^ that reveal how co-occurring systems of oppression interact in terms of power.^30^ Yet, the understanding of vulnerability, itself a nuanced concept, has been the subject of little scrutiny, particularly in the sex trafficking context.^24^ Therefore, we also employed a Taxonomy of Vulnerability to explore providers’ perceptions of vulnerabilities to domestic sex trafficking.^17^

## Methodology

This study on the perceptions of providers regarding vulnerabilities to sex trafficking employed a qualitative exploratory design and is part of a larger project exploring the knowledge, attitudes, and practices of physicians, nurses, and social workers in Ontario, Canada.^13,31,32^ Ethics approval was acquired from the Women’s College Hospital Research Ethics Board in September 2023 (REB #: 2023-0013-E).

### Study Setting

This study was conducted in Ontario, Canada’s most densely inhabited province. Data were collected from diverse healthcare providers working in five out of seven Ontario Health regions, Central west, Central east, Southwest, East, and Toronto, including urban, suburban, urban/suburban, rural, and remote locations.^33^ Healthcare in Ontario is delivered through a publicly funded system operating in these health regions. Individuals living in Ontario can access healthcare through hospital emergency departments, primary care services, and community clinics. Persons who are sex trafficked can access services and support, where available, through various government-funded and not-for-profit agencies such as Ontario’s 37 hospital-based Sexual Assault/Domestic Violence Treatment Centres.

### Recruitment

Study invitation emails were sent to a compiled list of healthcare organizations as well as individual professionals across the seven regions of Ontario. These emails included the ethics approval, the study description, and participation requirements such as the eligibility criteria, and availability to participate in a one-time interview. A similar flyer was shared on Twitter. Recruitment occurred from November 2022 to February 2023, using purposive and snowball sampling across all seven Ontario Health Regions, to ensure diversity in age, gender, ethnicity, profession, and professional development, and community. To ensure a diversity of perspectives, participants’ identities, social positions, and professional roles were documented as interviews were being conducted. We employed specific targeted recruitment strategies to capture a diverse sample, including recruiting from culturally specific healthcare organizations.

In accordance with qualitative research recommendations, recruitment ended when saturation was reached, that is, when no new information emerged from the interviews.^34,35^ Participants were required to be proficient in English, reside and practice in a healthcare setting in Ontario, and provide informed consent. Participants were included regardless of their experience with providing care to domestically sex-trafficked individuals. The research team chose this approach as providers may not be aware if they had cared for a person who was sex trafficked unless the individual chose to disclose this information.^13,31,32^ Interested individuals completed a sociodemographic questionnaire to confirm their eligibility. Consent forms detailing the study and confidentiality measures were reviewed with each participant. They were informed that all interview recordings would be destroyed five years after publication. Participants signed and returned the consent forms after having any of their concerns addressed.

### Participant Characteristics

This study of 31 participants included social workers (39%), physicians (29%), and nurses (32%); no participants withdrew at any point in time. The majority identified as women (84%), ranging in age from 25 to 69. Approximately, 18 participants identified as White. Additionally, three participants identified as South Asian, three as Black, and two as Jewish. The remaining participants self-identified as Indigenous, Chinese, Mestizo, Southeast Asian, and Southeast Asian/Irish, with one individual in each of these categories. Twenty-three participants were employed in urban areas, three in suburban, two in urban/suburban, two in rural areas, and one in a remote location.^13^ A total of 20 providers reported having prior experience providing direct care for someone who is/has been sex trafficked, while three had no experience and eight providers were unsure. Further, in self-rating their overall expertise in caring for individuals who have been victims of sex trafficking, one provider rated their expertise as very high, three rated it as high, twelve reported having moderate experience, twelve rated their experience as low, and three indicated they had no expertise at all.

### Data Collection

Interviews were originally conducted and recorded on Zoom, lasting 60 to 90 minutes. Participants selected a pseudonym, and interviewers ensured no other participants selected the same pseudonym. Consent was re-confirmed after introductions, and the remaining questions were addressed. The interview guide was developed through a review of the literature, as well as the research team’s professional and lived experiences in the field. Eleven open-ended, semi-structured questions were organized under 3 main headings: knowledge, attitudes, and practices related to domestic sex trafficking (Supplemental File 1 interview guide). The questions under the knowledge section focused on providers’ understanding of the definition of domestic sex trafficking and the sources they rely on to learn about the issue.^13,31,32^ For example, one question aimed at assessing participants’ knowledge asked, “Who do you think are the usual victims of sex trafficking?” The section on attitudes included questions regarding providers’ opinions and beliefs about who is likely to be a victim of sex trafficking.^13,31,32^ Finally, questions under the practices section focused on providers’ formal education and training on sex trafficking, as well as an assessment of providers’ roles in the context of caregiving.^13,31,32^ Additionally, some questions were adapted from the Human Trafficking Myths Scale.^31,36^ Specifically, the Human Trafficking Myths Scale informed questions related to gender and attitudes about sex trafficking.^36^ For instance, a question focused on providers’ knowledge, such as, “how is this the same or different from sex work?” was adapted from the myth, “if a person receives any kind of payment for sex, he or she is not being trafficked.”^36^ Further, under attitudes, the question, “who do you think are the typical victims of sex trafficking?” was rephrased from the myth, “normal-appearing, well-educated, middle-class individuals are not trafficked.”^36^ Additionally, the question, “are certain groups more likely to try to escape sex trafficking or seek help?” was adapted from the myth, “if someone did not want to be trafficked, he or she would leave the situation.”^36^ The guide was then pilot tested within the research team.

Audio transcripts were automatically generated, and field notes captured additional contextual information. All files were password protected and securely stored in an institutional drive. Subsequently, audio and video recordings were deleted from Zoom. Verified transcripts were anonymized and imported into Dedoose Management Software (Version 9.0.90, 2023). To thank the participants for their time and efforts, a $25 online gift card was provided.

### Data Analysis

The interviews were analyzed using the following six phases of Braun and Clarke’s Reflexive Thematic Analysis framework to ensure rigor and reliability. The six phases include; familiarization with the data, generating draft codes, developing initial themes, finalizing and defining themes, and manuscript development.^37^ During the first two phases, four team members familiarized themselves with four interviews by independently reading and re-reading the transcripts to generate deductive codes, followed by inductive codes and subthemes.^13,31,32^ Team members regularly discussed any differences in their coding by eliminating, refining, or merging codes as needed. To minimize bias, team members also engaged in reflexivity by continuously reflecting on and interpreting their field notes and memos. Throughout this process, we were aware of and discussed the intersection of our social positions, privileges, and perspectives and challenged each other when these seemed to result in biased interpretations. These exchanges often led to valuable insights about how each member’s work, research, or diverse lived experiences might have shaped their perspectives and, in turn, the development, deleting and finalizing of draft codes. For example, how team members situated themselves theoretically due to their previous training or professional experiences in qualitative research played a role in generating deductive and inductive codes. Additionally, the team continuously worked solely on participants’ meanings and not team member’s interpretations. The result was a preliminary code book.

**Figure 1.**
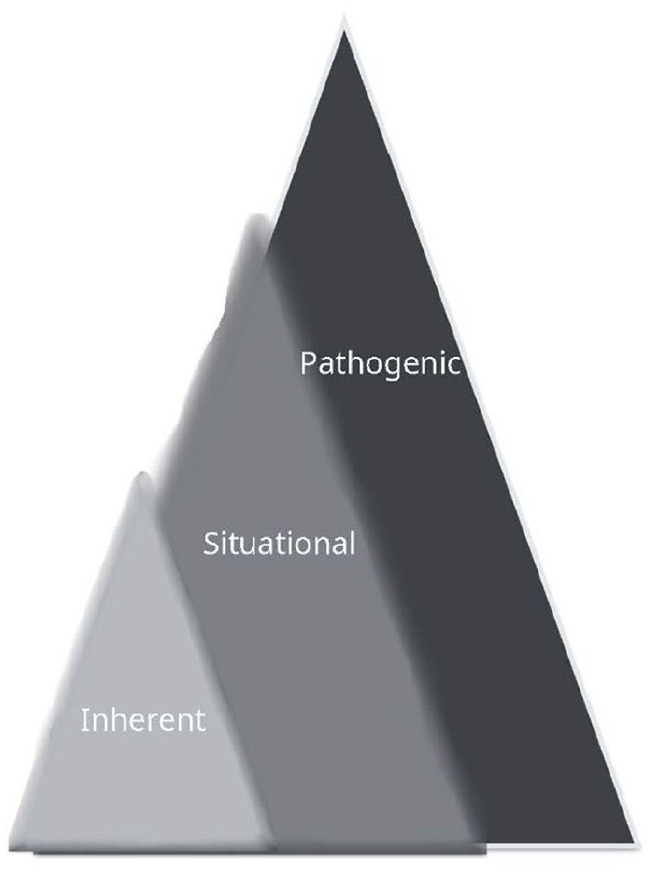
A modified Taxonomy of Vulnerability model. *Note.* This model captured the dynamic interplay among inherent, situational and pathogenic vulnerabilities which highlighted blurred boundaries of the perceptions of these vulnerabilities. While the Taxonomy of Vulnerability considers pathogenic as a subset of situational^17^ our study team modified the taxonomy to consider each source of vulnerability in a pyramidal model, with inherent vulnerabilities at the top considering they are universal to all humans, situational vulnerabilities includes both inherent and context, and pathogenic represents the intersection of inherent, situational, and pathogenic vulnerabilities.

Two members then used the preliminary code book to code two additional transcripts. The team discussed and reconciled any new codes and subthemes; the result was a final codebook. In phase three, the team focused on applying the codebook to the remaining transcripts. During phase four, our team reviewed the subthemes and differentiated them into the categories of vulnerability within the Taxonomy, using the definitions provided, with one minor modification.^17^ We separated pathogenic from situational vulnerabilities to comprise three categories: inherent, situational, and pathogenic^17^ (see Figure 1). For example, if providers referred to specific economic factors, their perception was classified as situational. When a provider described individuals as susceptible to sex trafficking due to abuse by those they depended on for care, this was categorized as pathogenic. As a result, we captured the dynamic interplay between the vulnerabilities in the subthemes to finalize the themes. We allocate supporting representative quotes to each theme in phase five. Finally, in phase six we drafted the manuscript.

## Results

Across participants’ perceptions regarding the vulnerabilities to sex trafficking, 3 themes were determined: (1) Traumatic history; (2) Social identities and relationships; and (3) Structural determinants. Within each theme, subthemes are organized by level of modified taxonomy (inherent, situational, and pathogenic) with supporting participant quotes.

### Theme 1: Traumatic History

In describing vulnerabilities to domestic sex trafficking, participants referenced the traumas persons who are sex trafficked may have experienced, as a result of unmet intrinsic human needs. Subthemes included: lack of support, coping with difficult life circumstances, and adverse childhood experiences.

#### Inherent Vulnerability

##### Lack of Social Support

Participants frequently cited an individual’s dependency on others for needs, that may not have been met, as a key source of vulnerability to domestic sex trafficking. Miss Spell (physician) cited “people who are isolated, people who have a great need” are vulnerable. Further, participants also highlighted unmet needs that were described as psychological, emotional, and physical needs such as a sense of belonging, love, care, and safety as inherent vulnerabilities. Corrine (social worker) expressed that “a young person or an individual doesn’t feel safe or have (a sense of) belonging, a sense of connection to the community. Or there’s a person who feels safe, [someone] in their life to confide in.” While inherent vulnerabilities may be related to the common human need for support, love and acceptance, there were other aspects of traumatic history that could be better understood as situational in character.

#### Situational Vulnerability

##### Lack of Familial Support

Participants also reported that individuals lacking familial support are isolated and lack parental guidance. Thus, these co-occurring inherent and situational vulnerabilities render them vulnerable to trafficking. Lisa (social worker) said, “Children predominantly from single parent households, or have both parents involved but [both] working a lot, so they’re isolated and on their own a lot.” Additionally, Sandra Smith (physician) similarly said, “So, people without, you know, strong social or family support. People with just like less, you know, fewer social supports, fewer kind of areas to seek help.” Finally, Sharon, (nurse) acknowledging that age created an additional vulnerability intersecting with situational vulnerabilities, added the following:

For a young person who is looking at self-determination and seeing expressions of love and different ways and adoration, and admiration that’s very attractive and so if you’re vulnerable to something like that, and also living in a household, that is chaotic. Maybe there isn’t a lot of affection or a lot of attachment there to begin with, that’s another circumstance of vulnerability.

##### Difficult Life Circumstances and Contexts

A few participants noted particular circumstances, such as substance use, that can exacerbate vulnerability to sex trafficking (Kalhaku, social worker; Sharon, nurse; Sarah, nurse). Although Sarah (nurse) highlighted that substance use by a parent can leave one vulnerable to isolation and thus, trafficking, other participants spoke of individuals relying on substances to alleviate the trauma experienced. Sharon (nurse) said the following:

We see a lot of substance use for people, [How in the world can you anticipate someone living in a dumpster without using], either they’re introduced to it by their trafficker, because it numbs away the experience, and maybe it’s prefaced as it’s not that bad, and then they become more dependent. It’s a vicious cycle.

#### Pathogenic Vulnerability

##### Adverse Childhood Experiences

Vulnerabilities that co-occur from the interaction of inherent and situational factors, leading to dysfunctional or abusive interpersonal and social relationships, and sociopolitical oppression or injustice, were classified as pathogenic. For example, some participants perceived that having unfulfilled needs at a younger age could lead to unsafe relationships with unequal power dynamics, increasing vulnerability to sex trafficking (Sharon, nurse; Corrine, social worker; Kalhaku, social worker; Jessica, social worker). Kalhaku (social worker) communicated that “They would be predisposed to conflict. . . [because] they’re looking for that love, and they may find it in a dysfunctional unhealthy person.” Moreover, the absence of parental figures was noted throughout participants’ perceptions. Kalhaku further adds that “Those that struggle the most do not have a healthy [relationship with their] father, or [he was] absent. The common theme is that they do not have a healthy father in their life.” Additionally, some participants drew connections between the trauma experienced by young individuals due to a lack of support and differential power dynamics and perceived such an intersection of vulnerabilities as susceptibility to trafficking. (Betty, nurse; Mako, nurse; Leslie, physician). As Mako noted, “They’re younger and they don’t have any support, and they find themselves homeless and someone is grooming them and taking advantage of them.”

Several participants reported experiences of previous or current abuse as a form of childhood trauma leading to vulnerability to sex trafficking. As Bugambilia (social worker) cited, “Young people who are living in homes where there is abuse.” Specific types of abuse, such as sexual, physical, and emotional abuse were mentioned by some participants (Julia, social worker; Miss Spell, physician; Kalhaku, social worker). Miss Spell (physician) expressed that “Early childhood experience in a general sense, and I think those who’ve had a history of physical, sexual, emotional abuse are at high risk.”

### Theme 2: Social Identities and Relationships

The theme of social identities and relationships was framed by participants’ perceptions of social identities as related to the vulnerabilities to domestic sex trafficking. Subthemes included differences in vulnerabilities according to gender, intersecting vulnerabilities based on identity, financial insecurity, and power dynamics of relationships.

#### Inherent Vulnerability

##### Differences in Vulnerabilities

The vulnerabilities inherent to the human condition are not indicative of the context in which they arise. Though women were perceived as more vulnerable, a few participants reported that males were also vulnerable to sex trafficking, while making no reference to context (Betsy Peacock, nurse; Sandra Smith, physician). As Sandra Smith (physician) noted, “I used to think there was more of a preponderance of females rather than males, but I think probably it’s equivalent or near equivalent.” Finally, being Indigenous was noted by several participants as a vulnerability to sex trafficking, also making no reference to context. As Bob (physician), noted, “I see mainly the Indigenous population; those are the cases that have come to my attention. There could be other people, I just don’t really know.”

#### Situational Vulnerability

##### Intersecting Vulnerabilities

Situational vulnerabilities are context-specific and have a relation with time, space or conditions, such as the performance of gender. For example, for some participants, being transgender was considered a vulnerability to sex trafficking within specific contexts (Hailey, nurse; Miss Spell, physician). Jane (social worker) stated, “I have also seen a chunk of individual transgender folk who were involved in that.” Some participants’ perceptions of vulnerability due to age intersecting with gender also extended to non-gender-conforming individuals (Zack, social worker; Jane, social worker). Zack (social worker) noted, “I would say, probably younger individuals and individuals who self-identify with the LBGTQ+ community are probably more likely to [be trafficked].” A few participants suggested the intersection of age, physical appearance, and social identity as a vulnerability to sex trafficking (Miss Spell, physician; Vinnie, nurse). As Miss Spell (physician) said:

I don’t know the age range, certainly adolescent, certainly women, probably mostly younger women who are seen as desirable. I think it’s in terms of the sex trade the majority would be women or females, people who identify as female and mostly younger.

Additionally, the converging of age and desirability as related to culture was also noted. Vinnie (nurse) stated that “[Young people] that are more exotic. If you’re from Canada and [exposed to mostly White people]. You might choose someone who is from a different culture. That might be more appealing.”

Similarly, participants often cited financial insecurity as a situational vulnerability, sometimes intersecting with age and gender. When individuals have a limited livelihood due to financial strain, participants perceived these individuals as vulnerable in the sense that they would do anything to survive. As Kerry (social worker) said, “People that are not able to sustain themselves financially in one way or another. They need financial support, so when a chance for a job comes, they’ll definitely run into it.” As Zack (social worker) noted, “[p]robably someone who is younger because, as a general rule, when you’re younger, you have less access to resources generally, so you’re more vulnerable financially.” Lastly, age, gender, and lack of education were noted to intersect with financial security. Mako (nurse) said, “Younger women and women who have less education, money [and] job security” are vulnerable to being sex trafficked. Thus, vulnerabilities to sex trafficking, inherent to being human intersected with situational stages of life.

#### Pathogenic Vulnerability

##### Power Dynamics of Relationships

Contextual vulnerabilities that raise ethical concerns due to an intersection with abuse and sociopolitical were positioned as pathogenic vulnerabilities. Participants perceived the power dynamics within specific relationships as a vulnerability to sex trafficking. Specific relationships cited by participants with inherent power imbalances ranged from familial, social acquaintances to complete strangers. As Ruby (social worker) pointed out:

The person recruiting is looking for someone that they can easily manipulate and control and make money from. The person who’s recruited might be in poverty, may lack in education, might be looking for a caregiver, a parental figure, might be wanting things like connection and intimacy, because they are alone or never had it growing up, might want to belong to a community, a group might be insecure.

Some participants noted further that persons experiencing mental health concerns or navigating a disability are vulnerable to sex trafficking through such power imbalances (Sharon, nurse; Sam, nurse; Ruby, social worker; John, physician). Sharon (nurse) said, “People navigating mental health chronically, I think, is another big population who are particularly vulnerable.” Sam (nurse) explained further:

We see those sometimes with mental health concerns where they don’t have support, and then their trafficker offers them something that sounds like it’s good support, and in the beginning. It is a positive situation in their eyes, and then it turns to the negative.

Regarding persons navigating disabilities, for some participants, susceptibility to sex trafficking stemmed from being physically vulnerable. Ruby (social worker) said “People with disabilities, people who appear vulnerable, you know when people are going out seeking or looking for someone to recruit,” are vulnerable to sex trafficking. However, Sharon (nurse) considered persons with learning disabilities as vulnerable, especially “people who are navigating [at a low] education scale, with not a lot of health literacy, not a lot of capacity, of knowledge.” The participants’ perceptions were not limited to certain disabilities and thus highlighted nuanced susceptibilities to sex trafficking.

### Theme 3: Structural Determinants

The intersection of vulnerabilities, noted by participants as the social determinants of health for marginalized and racialized individuals or groups differentiated the theme of structural determinants. Subthemes included groups who are marginalized and/or racialized, physically isolated environments, stigma and common stereotypes.

#### Pathogenic Vulnerability

##### Manifestations of Marginalization and/or Racialization

Some participants’ perceptions highlighted issues of marginalization and racialization. As noted by Bugambilia (social worker), individuals perceived as vulnerable include “Racialized individuals, Black, Indigenous, and Latino. I will say mainly those groups” who are overrepresented among those vulnerable to sex trafficking. However, another participant Sandra Smith (physician), noted, “I used to work with First Nation communities, and I would say like learned helplessness is a big thing.” A few participants noted being marginalized due to gender and racialized identity as a vulnerability to sex trafficking (Lisa, social worker; John, physician; Miss Spell, physician). Miss Spell (physician) said:

If we look at the meso and micro, gender inequity plays a role. Holding on to and expanding our position of equity as gendered women, those who identify as women, or who identify as bisexual or queer, or non-gendered, and anybody who is outside of the patriarchal norm of male. That would include racialized populations. We need to continue to drive for equity and preserve it.

Further, Helen (physician) stated, “In Canada, at least, it is people from marginalized groups who are overrepresented as people who are trafficked. Indigenous, Trans, people of racialized backgrounds.” Another participant, who also shared the perception that anyone viewed outside of dominant understandings of gender and race/ethnicity is vulnerable to sex trafficking, highlighted the historically derived intersecting vulnerabilities. Lisa (social worker) said, “In terms of ethnic group, I would imagine persons of color, whether that be like from the South Asian community, East Asian, or African American. [Communities that are] fetishized by Western society.” Marginalization based on socioeconomic status, age, and use of substances were discerned as intersecting vulnerabilities with racialized identities by a few participants. Sandra Smith (physician) illustrated some of these vulnerabilities that could lead to recruitment:

Yeah, so socially isolated, racially, impoverished. Perhaps tied in a drug and alcohol addiction, because they might be a bit more dependent upon that substance and then being more lured and groomed to get that substance, by a trafficker, I guess.

##### Physically Isolated Environments

Some participants’ perceptions of precarity to sex trafficking also included living in an isolated environment (Sarah, nurse; Sharon, nurse; Megan, nurse; Corrine, social worker). This perceived vulnerability highlighted the intersection of inherent, situational, and structural determinants. Sharon (nurse) described:

I think environmental vulnerability in terms of different areas of Canada that are particularly isolated. [If] we think of places in our Northern communities that are very isolated regarding transportation. If you do not have access to [services], how would you ever get out of that circumstance if you’re in a small community? Who do you ask for help if you’re trafficker, maybe also the person who you’re supposed to ask for help.

Participants noted that these intersecting vulnerabilities left individuals subject to power imbalances in relationships, especially with traffickers who can mitigate the challenges linked to living in an isolated environment. Corrine (social worker) explained, “Traffickers would tend to weed out those individuals to see who’s vulnerable, and a lot of times it could be in isolated areas.”

##### Stigma and Common Stereotypes

A few participants who specifically noted the role of stigma and commonly held stereotypes simultaneously challenged and underpinned perceptions of vulnerabilities to sex trafficking. Corrine (social worker) noted:

A lot of times when we have an image of someone that is being trafficked, we might have that image of someone that’s homeless or street-involved. I think that’s still a challenge right now is that we still have that perception that it’s all [people] shelters that are involved. I think we always go back to those stereotypes and stigmas that we have.

Stigma alone as a vulnerability to trafficking was further described by Sharon (nurse), who questioned:

How do you identify that you need help when you’ve been already stigmatized as someone who doesn’t need help. You’re not in a place, [due to] your own mental wellness, that you’re able to identify what has happened, what’s going on, and that you need to help. Right your focus is survival.

It is worth noting that a few participants reflected on their personal assumptions on who is vulnerable to trafficking and conveyed that it could be anyone (Sharon, nurse; Megan, nurse; Claire, social worker; Corrine, social worker; Rachel, physician). Corrine (social worker) cautioned that “It’s easy to [assume] that only those who are street-involved are at risk. I always have to caution myself because it’s easy to just see one small percentage of the population.” Megan (nurse) and Claire (social worker) specified that it may not just be young women who are vulnerable to sex trafficking, as opposed to their initial beliefs. Sharon (nurse) further perceived the complex blurring of unmet needs and marginalization by the structural determinants as a vulnerability to sex trafficking:

To be honest. I think anyone can be vulnerable to it. I don’t think it parlays a particular demographic, though I think that people who do have less capacity in society are more vulnerable to being victimized. We often see people who are navigating adolescence go through that, but I think it’s anyone who is in a vulnerable environmental and economic situation, which is to be honest, it could be anyone at any particular point.

## Discussion

In what we believe to be the first time, we applied critical social theory, intersectionality, and a modified Taxonomy of Vulnerability framework to understand providers’ perceptions of vulnerability to domestic sex trafficking. This study’s findings confirm previous research indicating that providers consider a “traumatic history”—including experiences of physical, emotional, or sexual abuse—to be a significant indicator of vulnerability.^25,38-40^ This study contributes to existing scholarship on domestic sex trafficking by highlighting providers’ limited understanding of how other factors, such as “social identities and relationships” and “structural determinants” affect those deemed vulnerable to sex trafficking. Finally, this study emphasizes the need for critical approaches in provider training to better understand the complex ways larger systems contribute to susceptibility to domestic sex trafficking questioning the notion that vulnerability to sex trafficking is universal. Together, this information can now be integrated into understandings of vulnerability with implications for necessary, broader social/societal change.

Across all three themes, providers consistently identified being female as a vulnerability to domestic sex trafficking. Some providers went on to also note that being female was associated with having less power in various relationships (eg, intimate partner, familial, friendship), or a lower socioeconomic status aligning with previous research.^38,39^ However, these providers did not recognize the ways in which social, economic, and educational systems and “laws, policies and practices of states” (p. 41)^41^ serve to perpetuate gender inequalities. For example, providers did not refer to the underfunding of education in marginalized communities the ongoing legacy of segregation of Indigenous peoples; or how socio-economic policies chronically underfund services, shelters, and public housing for women perpetuating cycles of homelessness, exploitation, violence, and poverty in the lives of women, when they described marginalized and racialized individuals or groups as vulnerable to domestic sex trafficking.^40,42^ Some providers in this study acknowledged the biases, stereotypes, and misconceptions they hold, which informed who they considered vulnerable to sex trafficking. Consistent with previous research, these providers did link their biases to the information they read, their observations, and assumptions—ultimately impacting their ability to identify persons who are sex trafficked.^13,25,32,39,43^ To ensure providers are better equipped to provide appropriate and sensitive care, the development of a more nuanced understanding of vulnerabilities to domestic sex trafficking is essential. Recognizing that vulnerabilities are not necessarily implicit to being female but are instead the result of systems’ intentional socialization processes,^21^ the same systems in which providers have been socialized. What is needed, as recent work has also stressed, is a “critically conscious approach” (p. 8),^43^ (p. 12)^44^ to provider training, one that highlights the processes that lead to the over-representation of females in sex trafficking. The initial implication of this approach is the enhancement of healthcare providers’ practices for identifying persons who are sex trafficked as they learn to recognize the role of systems in perpetuating vulnerability. Secondly, provider training informed by a “critically conscious approach” (p. 8),^43^ (p. 12)^44^ also empowers them to counter prevailing narratives of vulnerability. Especially narratives that represent certain individuals as deficient, dependent, and passive and place the responsibility for change and resilience solely on the individual.^20,40,44,45^

Few providers referenced the intersections of being female with other sociodemographic characteristics associated with vulnerability to domestic sex trafficking, such as, being Indigenous, Asian, or of African descent. However, these providers did not link their perceptions of these intersections further to the complex ways larger systems have perpetuated certain individual’s or s’ and groups’ marginalization and inequitable status.^46^ In turn, this oversimplification limits our understanding of the underlying causes of this vulnerability. Overlooking these larger forces and structures may reduce the complex systemic issues that contribute to vulnerability to domestic sex trafficking into racial profiles and narratives.^47^ In turn, this oversimplification limits our understanding of the underlying causes of this vulnerability. Further, when we operationalize these narratives within the context of healthcare, we may view the issue of domestic sex trafficking as inherent, with “the problem [stemming] with the Other and their culture” (p. 623).^48^ Such a perspective leads to policies and care practices that unjustly restrict or deny access to healthcare.

Some healthcare providers perceived vulnerability to sex trafficking as universal to all humans. The concept of universality in the Taxonomy of Vulnerability, characterized as “inherent” may be considered as a way to improve services and supports for persons who are sex trafficked. However, it can inadvertently reinforce inequity, posing a challenge to critical social theory and intersectionality. By adopting an “over-inclusive approach” (p. 21)^49^ to vulnerability, we overlook the need to consider the complexities of individual circumstances and how specific populations experience unique vulnerabilities due to historical and systemic oppression related to “power, privilege, and identity” (p. 5).^22,50,51^ As some have noted, this approach “fail[s] to be action-guiding” (p. 26).^49^ Additionally, this perspective has significant policy implications, particularly concerning the adequate funding of services, access to suitable services, and the types of services provided. Other researchers have challenged the category of inherent vulnerabilities^49^ as “independent of time, space, and conditions” (p. 759).^23^ For example, Luna proposed the concept of “layers of vulnerability” through which contexts are dynamic and change over time (p. 130).^50^ Rogers suggested beginning with a definition of vulnerability that encompasses its universality and how these vulnerabilities manifest differently in the lives of all individuals.^49^ These conceptualizations may provide a better way to guide further research in understanding of vulnerabilities to domestic sex trafficking as, in our study, there were some healthcare providers who recognized or understood vulnerability to be a more complex phenomenon, linking their perceptions of inherent sources to other situational and pathogenic factors occurring simultaneously.

### Limitations

While this study includes healthcare providers from various workplaces, professional backgrounds, ages, and genders, the results may not be generalized to represent the views of all healthcare providers. We recruited a sample of healthcare providers that reflected the racial and ethnic diversity of Ontario, which may differ from other regions of Canada. Future research should include persons with lived experiences as they are best positioned to describe what made them vulnerable to domestic sex trafficking. Furthermore, exploring providers’ perceptions regarding susceptibilities to domestic sex trafficking across various professions, geographic locales, types of services, as well as the potential impact of previous formal and informal education and or training on providers’ perceptions would help further our understanding of how to tailor training to specific needs and conditions. Despite these limitations, the study highlights the need for a more nuanced understanding of vulnerabilities related to domestic sex trafficking, explores a relatively unexplored area, and paves the way for future research. A more nuanced understanding of the vulnerabilities that can increase susceptibility to domestic sex trafficking is essential if we wish to see domestically sex trafficked persons receive considerate, appropriate care, and meaningful support from their providers and policy decision-makers. Further, this knowledge may also help to counter prevailing harmful narratives that serve to perpetuate harmful, stigmatizing attitudes.

## Conclusion

Utilizing a critical social theory and intersectionality framework and applying a modified Taxonomy of Vulnerability highlighted some providers’ lack of awareness of the role that systems play in perpetuating intersecting vulnerabilities in people’s lives. As a result, there may need to be a deliberate fostering of a “critical conscious approach” (p. 8),^43^ (p. 12)^44^ within provider training-specific to understanding the contextual roots of vulnerability and the social control imposed by larger systems that impart a false hierarchy of human value.^41^ An intersectional perspective on broader influences prevents oversimplifying complex systemic issues that perpetuate various vulnerabilities to sex trafficking into harmful racial profiles or a discourse of the universality of vulnerability. When these profiles and narratives are operationalized in a culture of care, associated policies and practices fail to consider the importance of the individual domestically sex trafficked in larger contexts.

## Supplemental Material

sj-docx-1-his-10.1177_11786329251348295 – Supplemental material for Healthcare Providers’ Perceptions of Vulnerability to Domestic Sex Trafficking in Ontario: A Qualitative Study Supplemental material, sj-docx-1-his-10.1177_11786329251348295 for Healthcare Providers’ Perceptions of Vulnerability to Domestic Sex Trafficking in Ontario: A Qualitative Study by Corinne Rogers, Soumyaa Veerakumar Subramanium, Rhonelle Bruder, Robin Mason and Janice Du Mont in Health Services Insights

sj-docx-2-his-10.1177_11786329251348295 – Supplemental material for Healthcare Providers’ Perceptions of Vulnerability to Domestic Sex Trafficking in Ontario: A Qualitative Study Supplemental material, sj-docx-2-his-10.1177_11786329251348295 for Healthcare Providers’ Perceptions of Vulnerability to Domestic Sex Trafficking in Ontario: A Qualitative Study by Corinne Rogers, Soumyaa Veerakumar Subramanium, Rhonelle Bruder, Robin Mason and Janice Du Mont in Health Services Insights
