# Supplementary material for: Healthcare Providers’ Perceptions of Vulnerability to Domestic Sex Trafficking in Ontario: A Qualitative Study
Source: Health Serv Insights. 2025 Jun 24;18:11786329251348295. doi: 10.1177/11786329251348295 (PMC12188056; doi:10.1177/11786329251348295)
Supplement: sj-docx-1-his-10.1177_11786329251348295 – Supplemental material for Healthcare Providers’ Perceptions of Vulnerability to Domestic Sex Trafficking in Ontario: A Qualitative Study [file sj-docx-1-his-10.1177_11786329251348295.docx]

**Supplemental Material**

**Semi-Structured Interview Guide**

**Q1 (Knowledge)**: In your own words, what is sex trafficking?

*Prompt*: What does domestic sex trafficking include?

*Prompt*: Where did you learn this information (e.g., popular culture)?

*Prompt:* How is this the same/different from sex work?

**Q2 (Knowledge + Attitudes)**:  Who do you think are the usual victims of sex trafficking?

*Prompt*: How do you think individuals come to be sex trafficked (who is the trafficker)?

*Prompt*: Why might people who are sex trafficked remain in sex trafficking?

*Prompt*: Are certain groups more likely to try to escape sex trafficking or seek help?

*Prompt*: What social and other circumstances do you think affect whether someone becomes sex trafficked? Can you tell me a bit about those?

**Q3 (Knowledge + Practices):** What formal education or training on sex trafficking have you had (if any; e.g., 1 session vs. a course)?

*Prompt*: How long was the training?

*Prompt:* Was the training online or in-person?

*Prompt:* What did the training cover (e.g., who is sex trafficked, indicators sex trafficking)?

*Prompt:* Who led the training?

*Prompt:* What do you think was missing from the training?

*Prompt:* Have you been able to apply this training in practice? If so, how?

**Q4 (Attitudes + Practices)**: Would you approach or do anything differently with a (suspected) person who has been sex trafficked versus other patients?

*Prompt*: How do you think your own social identity (race, gender, class, migration status, etc.) might affect the ways you would help/connect with people who have been sex trafficked?

**Q5 (Knowledge + Attitudes + Practices)**: How would you identify someone who is being sex trafficked?

*Prompt*: What are the red flags/indicators (e.g., What might you see or hear that would indicate that someone is being trafficked, clues from the person accompanying the patient)?

*Prompt:* What are the challenges in identifying someone who has been trafficked?

**Q6 (Practices):** How would you support a patient who disclosed being (or who you suspected had been) sex trafficked?

*Prompt:* What do you think facilitated/got in the way of disclosure?

*Prompt:* What were any physical/mental health symptoms or social concerns (e.g., stable housing, community supports)?

*Prompt*: What did you do about these symptoms/concerns?

*Prompt:* What was the outcome for the patient?

Prompt: What could have changed/improved the outcome for the patient?

**Q7 (Attitudes + Practices)**: What do you believe that patients who have been sex trafficked need most in general and also from you specifically as a health care provider? Why?

*Prompt*: In what ways are you able to provide that? If not, what do you normally do?

*Prompt*: Do particular individuals (or sub-groups) need/ask for more or less support? Do you feel confident providing support to these individuals?

**Q8 (Practices)**: What challenges stand in the way of you being able to provide the patient with appropriate support?

*Prompt*: Is there anything that would make it easier to provide patients with the care they express needing/you think they need?

*Prompt:* Are there supports at the organizational or departmental level that could improve your ability to provide care?

**Q9 (Practices):** What guidelines or protocols do you have to follow when caring for a person who has been sex trafficked?

*Prompt*: What kind of documentation do you need to fill out or complete about the visit (e.g., medical history, mental health history)?

*Prompt:* Does the patient’s sex trafficking status go on record?

**Q10 (Practices):** What kind of follow up or longer-term care are you able to provide to (suspected) patients who have been sex trafficked?

*Prompt:* Would there be any measures of safety you might implement?

*Prompt:* If you do not offer continued support, what kind of support do you offer?

*Prompt*: Who else might you involve in the care (in your organization or external to it)?

*Prompt*: What referral do you most commonly make? Why?

**Q11 (Attitudes + Practices)**: If any, what local resources are you aware of for patients who have been sex trafficked?

*Prompt*: Tell me about them (culturally specific or otherwise).

*Prompt*: Are any of these advocacy based? Do you participate in any related forms of advocacy?

*Prompt:* Are there any resources missing you think would help better support your patients?
